# Expression of FKBP prolyl isomerase 5 gene in tissues of muscovy duck at different growth stages and its association with muscovy duck weight

**DOI:** 10.5713/ab.20.0649

**Published:** 2021-06-23

**Authors:** Zhigang Hu, Liyan Ge, Huilin Zhang, Xiaolin Liu

**Affiliations:** 1 College of Animal Science and Technology, Northwest A&F University, Yangling, Shaanxi 712100, China

**Keywords:** Duck, FKBP Prolyl Isomerase 5 (*FKBP5*), Gene Expression, Single Nucleotide Polymorphism, Weight

## Abstract

**Objective:**

FKBP prolyl isomerase 5 (FKBP5) has been shown to play an important role in metabolically active tissues such as skeletal muscle. However, the expression of *FKBP5* in Muscovy duck tissues and its association with body weight are still unclear.

**Methods:**

In this study, real-time quantitative polymerase chain reaction was used to detect the expression of *FKBP5* in different tissues of Muscovy duck at different growth stages. Further, single nucleotide polymorphisms (SNPs) were detected in the exon region of *FKBP5* and were combined analyzed with the body weight of 334 Muscovy ducks.

**Results:**

*FKBP5* was highly expressed in various tissues of Muscovy duck at days 17, 19, 21, 24, and 27 of embryonic development. In addition, the expression of *FKBP5* in the tissues of female adult Muscovy ducks was higher than that of male Muscovy ducks. Besides, an association analysis indicated that 3 SNPs were related to body weight trait. At the g.4819252 A>G, the body weight of AG genotype was significantly higher than that of the AA and the GG genotype. At the g.4821390 G>A, the genotype GA was extremely significantly related to body weight. At the g.4830622 T>G, the body weight of TT was significantly higher than GG and TG.

**Conclusion:**

These findings indicate the possible effects of expression levels in various tissues and the SNPs of *FKBP5* on Muscovy duck body weight trait. *FKBP5* could be used as molecular marker for muscle development trait using early marker-assisted selection of Muscovy ducks.

## INTRODUCTION

Skeletal muscle, which accounts for 50% to 60% of the total weight, is one of the major tissues involved in regulating metabolism, movement, and strength [1]. In addition, skeletal muscle forms a network that regulates the function of distal organs and skeletal muscle itself by releasing various types of actin [2]. In livestock production, muscle yield is one of the most important factors in determining the economic value [3]. Skeletal muscle mass and fiber size are regulated in response to changes in animal activity, disease and aging through many genes, transcription factors or non-coding RNAs (lncRNA or circRNA) [4]. Skeletal muscle development is a rigorous procedural process that goes through several periods, i) Differentiation of muscle precursor cells from the somite; ii) The muscle precursor cells proliferate and differentiate into myoblasts; iii) Myoblasts fusion into myotubes; iv) Myotubes form muscle fibers [5,6]. Besides, at the late embryonic stage, myoblasts stop the cell cycle and fuse to form myotubes.

FK506 binding proteins (FKBPs) are multi-functional proteins that are highly conserved between species. They are abundantly expressed in cells and belong to members of the immunoaffinity protein family [7]. The molecular weight of FKBPs range from 12 kDa to 135 kDa. FKBP51, also known as FKBP5, has a molecular weight of 51 kDa, is involved in regulation of immune and basic protein folding and transport, and it is a member of the immunoaffinity protein family. The main research areas of FKBP51 include mental illness, anxiety, and depression [8,9], which may be due to the pivotal role of FKBP51 in the hypothalamic-pituitary-adrenal axis (HPA-axis) [10]. The HPA axis has a feedback control system that regulates the release of glucocorticoids [11], and FKBP51 has its ability to modulate glucocorticoid receptor (GR) sensitivity and HPA axis function [12,13]. FKBP51, as part of the heat shock protein 90 (Hsp90) steroid receptor complex, plays an important role in regulating the steroid hormone receptor (GR). Glucocorticoids induce expression of *FKBP5* at mRNA and protein levels as part of an intracellular negative feedback loop that regulates GR activity [14]. Moreover, *FKBP5* also plays an important role in metabolic regulation, such as inhibiting AKT [15] and mammalian target of rapamycin signal regulated kinases, and mediating autophagy [16], which is a catabolic process that maintains homeostasis, and is associated with a metabolic phenotype. *FKBP5* is confirmed to be most expressed in human skeletal muscle and adipose tissue in all study tissues [17], and increased *FKBP5* expression in omental adipose tissue is associated with insulin resistance [18]. In mice, the expression of *FKBP5* in medial hypothalamus (the brain area that regulating satiety and body weight) is induced by a high-fat diet and is positively correlated with stress-induced weight gain [19]. Besides, compared to wild-type mice, mice lacking *FKBP5* gene lose weight and are resistant to diet-induced obesity [20]. Some studies have believed that *FKBP5* may affect body weight and metabolism through multiple pathways and actions [21]. Although the mechanisms of these effects remain unclear, studies to date have agreed that up-regulation of *FKBP5* is associated with weight gain and negative metabolism.

*FKBP5* is also involved in NF-κB and Akt signaling pathways and it regulates the development of osteoclasts, survival, and activation of bone resorption. Lu et al [22] found that the novel mutation (c.163G>C) in *FKBP5* gene was associated with Paget’s disease of bone (PDB), and the mutation promoted osteoclast differentiation and bone resorption activity, both of which were major defect in PDB development. Hartmann et al [23] found that the excess body weight of carriers of the *FKBP5* rs1360780 polymorphism T allele was nearly 20% less than that of homozygous carriers of the C allele, and overall weight reduced 10% at 26 weeks of follow-up. Yeo et al [24] showed that changes in the rs1360780 locus could influence the expression of *FKBP5*, which affected the expression of other glucocorticoid-regulated genes. Therefore, *FKBP5* is speculated to be a potential gene that regulates muscle development.

Muscovy duck, also known as red-billed goose or musk duck, is native to the tropical regions of Central and South America. It was introduced to China more than 250 years ago. Muscovy duck has the advantages of large body size, rapid growth, resistance to rough feeding, easy fattening, delicious meat, and good liver performance. Currently, there are few reports on *FKBP5* gene in Muscovy ducks. Therefore, the expression patterns of *FKBP5* in Muscovy duck tissues at different growth stages were first studied, and then the association between Muscovy duck *FKBP5* single nucleotide polymorphisms (SNPs) and body weight was used to determine whether *FKBP5* could be used as a candidate gene for Muscovy duck growth.

## MATERIALS AND METHODS

### Ethics statement

All animal procedures were performed according to guidelines provided by the China Council on Animal Care, and the protocols were approved by the Experimental Animal Management Committee (EAMC) of Northwest A&F University.

### Animals and sample extractions

The eggs of Muscovy duck with similar size and weight were incubated in accordance with the incubation procedure after disinfection. Eight embryo eggs were randomly selected at the 17 d (E17d), 19 d (E19d), 21 d (E21d), 24 d (E24d), 27 d (E27d), 31 d (E31d), and 34 d (E34d) during the incubation period, respectively. Heart, liver, lung, kidney, breast muscle, and leg muscle were separated for RNA extraction. Similarly, 6-month-old Muscovy ducks were randomly selected (4 males and 4 females), and the tissues containing heart, liver, spleen, lung, kidney, breast muscle, leg muscle and testis/ovary were quickly isolated for RNA extraction after slaughter. Besides, a total of 344 Muscovy ducks (280 days old and unrelated for at least three generations) were randomly selected for blood sampling. Body weight data of Muscovy duck was recorded for association studies. Adult Muscovy ducks were raised under the same environmental conditions and had free access to feed and water (Supplementary Table S1). All Muscovy ducks and eggs were purchased from Anda Farm in Tongguan County, Shaanxi Province, China.

### RNA extraction and cDNA synthesis

RNA was isolated from different tissues of embryonic and 6-month-old Muscovy ducks using TRIzol Reagent (CWBIO, Taizhou, Jiangsu, China) according to the manufacturer’s instructions. The extracted RNA was detected by 1% agarose gel electrophoresis and NanoDrop 2000 (Thermo, Waltham, MA, USA) for quality and concentration, respectively. The first strand cDNA was synthesized from purified RNA by reverse transcription kit (abm, Richmond, BC, Canada) according to the manufacturer’s protocol.

### Tissue expression analysis of *FKBP5* gene in Muscovy duck

Spanned exon primers were designed by primer premier 5.0 based on the mRNA sequence of duck *FKBP5* gene (GenBank accession number: NC_040072.1) and *β-actin* gene (GenBank accession number: NC_040060.1) from GenBank (www.ncbi.nlm.nih.gov/) (Table 1). The relative expression of target gene was normalized against the internal control gene, *β-actin*. To determine the real-time quantitative polymerase chain reaction (qRT-PCR) efficiency of target and internal control genes, tenfold serial dilutions (10^−1^ to 10^−5^) of cDNA were produced and assayed in triplicate to yield standard curves. The identity of the amplified products was also confirmed by sequencing. The qRT-PCR reactions were performed in a total volume of 10 μL including 5 μL 2×TransStart Tip Green qPCR SuperMix (Transgen, Beijing, China), 0.2 μL of each primer (10 μM), 0.8 μL cDNA (400 ng/μL) and 3.8 μL ddH_2_O. Each cDNA sample was then analyzed in triplicate using EcoRT48 system (OSA, London, UK). The optimum thermal cycling conditions consisted of 95°C for 30 s, followed by 40 cycles of 95°C for 5 s, 60°C for 30 s, then 95°C for 15 s, 55°C for 15 s, 95°C for 15s. The relative expression level of *FKBP5* was calculated by 2^−ΔΔCt^ method. All data were subjected to one-way analysis of variance among different tissues.

### DNA extraction and polymerase chain reaction condition

Genomic DNA from Muscovy duck blood was extracted according to the phenol chloroform protocol, then DNA concentration and quality were measured by NanoDrop 2000 and 1% agarose gel electrophoresis, respectively. Finally, the genomic DNA was diluted to 80 ng/μL for storeage at −20°C. According to the nucleotide sequence of duck *FKBP5* gene (GenBank accession number NC_040072.1), 9 pairs of PCR primers (Table 1) were designed using primer premier 5.0 software. Each amplification reaction was carried out in a final volume of 15 μL reaction mixture containing 7.5 μL 2×Taq PCR Mix (CWBIO, China), 0.8 μL genomic DNA, 0.4 μL of each primer (10 μM), 5.9 μL ddH_2_O. The reactions were performed with the protocol consisting of initial denaturation at 94°C for 5 min, followed by 34 cycles of 94°C for 30 s, 30 s at the annealing temperature, and 60 s at 72°C, ending with a final extension at 72°C for 10 min.

### DNA pool sequencing and polymerase chain reaction-restriction fragment length polymorphism

To determine the mutation sites in the Muscovy duck *FKBP5* gene, 30 individual DNA samples were randomly selected to mix for a DNA pool. Subsequently, PCR amplifications were performed on two DNA pools and three random DNA individuals according to the above reaction conditions and reaction procedures. The target fragments were sequenced after verification by 1.5% agarose gel electrophoresis. Finally, the sequences were imported into the Chromas 1.62 for SNPs detection.

According to the location of mutation site, the restriction enzymes were selected for genotyping (Table 1). The digested reaction was carried out in 10 μL reactions containing 6 μL PCR product, 0.2 μL restriction enzyme (Takara, Tokyo, Japan or Thermo Scientific, USA), 1 μL 10×Buffer, 2.8 μL ddH_2_O, then it was reacted for 3 h after well mixed. The digested fragments were detected by 3% agarose gel electrophoresis.

### Data analyses

The allele frequency, genotype frequency, genotype frequency distribution, χ^2^ independence test, and genetic variation index, the gene heterozygosity (He), the effective allele numbers (Ne) and the relationship between the genotypes and the body weight of Muscovy duck were statistically analyzed. And polymorphism information content (PIC) was calculated by an online tool (www.msrcall.com). Association analysis of SNP and body weight was done using SPSS 20.0. The haplotype analysis was performed using Haploview (http://analysis.biox.cn/myAnalysis.php) [25].

The following general linear model was used for association test: Y_ijk_ = μ+M_i_+G_j_+F_x_+ e_ijk_, where Y_ijk_, the observed value; μ, the overall mean of individual traits; M_i_, the fixed effect of slaughtering age; G_j_, the fixed effect of genotype; F_x_, the fixed effect of sex; e_ijk_, the random error.

## RESULTS

### Tissue expression analysis of Muscovy duck *FKBP5*

**Figure 1:**
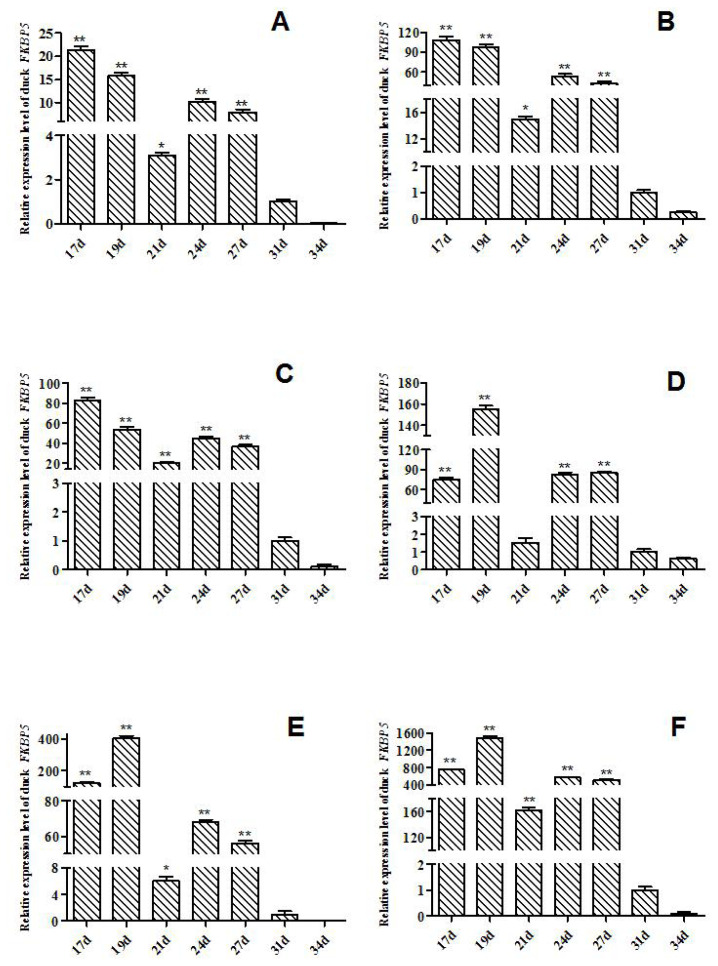
Expression of *FKBP5* gene at embryonic stages of Muscovy duck in various tissues. (A) Expression of *FKBP5* gene in heart. (B) Expression of *FKBP5* gene in liver. (C) Expression of *FKBP5* gene in lung. (D) Expression of *FKBP5* gene in kidney. (E) Expression of *FKBP5* gene in breast muscle. (F) Expression of *FKBP5* gene in leg muscle. The data were normalized to the expression of *FKBP5* on day 31 of incubation and calculated by using 2^−ΔΔCt^. *β-actin* was measured as the reference gene. Columns are represented as mean±standard deviation for three independent experiments performed in triplicate. *FKBP5*, FKBP prolyl isomerase 5. * Indicates that the difference is significant (p<0.05), ** indicates that the difference is extremely significant (p<0.01).

The expression levels of *FKBP5* gene in Muscovy ducks at different hatching stages were detected by qRT-PCR (Figure 1). In heart of Muscovy duck, the relative expression of *FKBP5* gene in E17d, E19d, E21d, E24d, and E27d was significant or extremely significantly higher than that of E31d (p<0.01 or p<0.05, Figure 1A). The qRT-PCR results showed that the relative expression trends of *FKBP5* in liver and lung were similar to that in heart, that is, the expression of *FKBP5* in E17d, E19d, E21d, E24d, and E27d were significant or extremely significantly higher than that of E31d (p<0.01 or p<0.05, Figure 1B, 1C). Besides, the expression trends in kidney and breast muscle were similar to leg muscle, where the expressions in kidney at the day 17, 19, 24, 27 of embryonic were extremely significantly higher than that of E31d (p<0.01, Figure 1D), and compared with the expression in E31d, *FKBP5* expression was significantly or extremely significantly higher in breast muscle in E17d, E19d, E21d, E24d, and E27d (p<0.01 or p<0.05, Figure 1E). *FKBP5* expressions in leg muscle in E17d, E19d, E21d, E24d, and E27d were significantly higher than that of E31d (p<0.01, Figure 1F). In all tissues, the expression levels of *FKBP5* in E34d were lower than that in E31d, but the differences were not significant (p>0.05).

**Figure 2:**
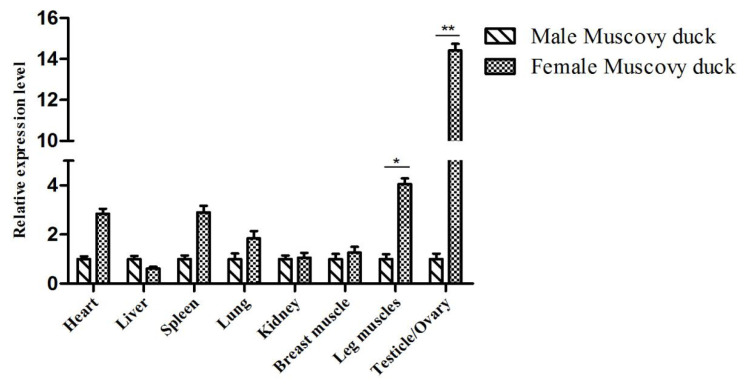
Expression of *FKBP5* gene in various tissues of 6-month-old Muscovy duck. The data were normalized to the expression of *FKBP5* in male Muscovy duck and calculated by using 2^−ΔΔCt^. *β-actin* was measured as the reference gene. Each Column represented the mean±standard deviation of three independent experiments which were performed in triplicate. *FKBP5*, FKBP prolyl isomerase 5. * Indicates that the difference is significant (p<0.05), ** indicates that the difference is extremely significant (p<0.01).

To determine the expression levels of duck *FKBP5* gene in different genders, 6-month-old male and female Muscovy ducks were selected as research objects. The expression of *FKBP5* gene in the tissues of female ducks was higher than male ducks’ (except liver), and in leg muscle of female ducks, the expression of *FKBP5* gene was significantly higher than that of the male Muscovy ducks (p<0.05). In addition, the expression of *FKBP5* gene in ovary was significantly higher than that in testis (p<0.01, Figure 2).

### Analysis of single nucleotide polymorphisms in *FKBP5* gene

**Figure 3:**
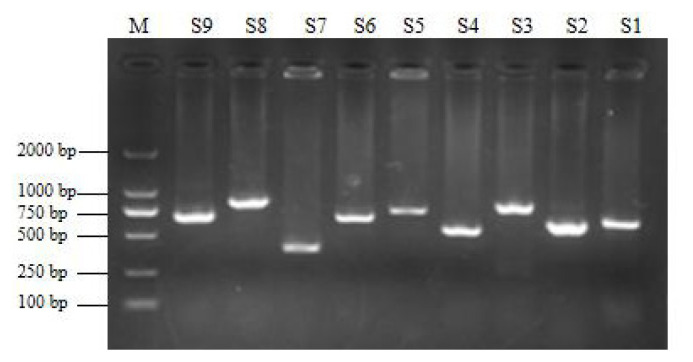
The amplification of *FKBP5* gene. The fragments of S1–S9 were 634 bp, 609 bp, 777 bp, 541 bp, 717 bp, 655 bp, 387 bp, 803 bp, 648 bp, respectively. Lane M was DNA DM 2000 Marker. *FKBP5*, FKBP prolyl isomerase 5.

**Figure 4:**
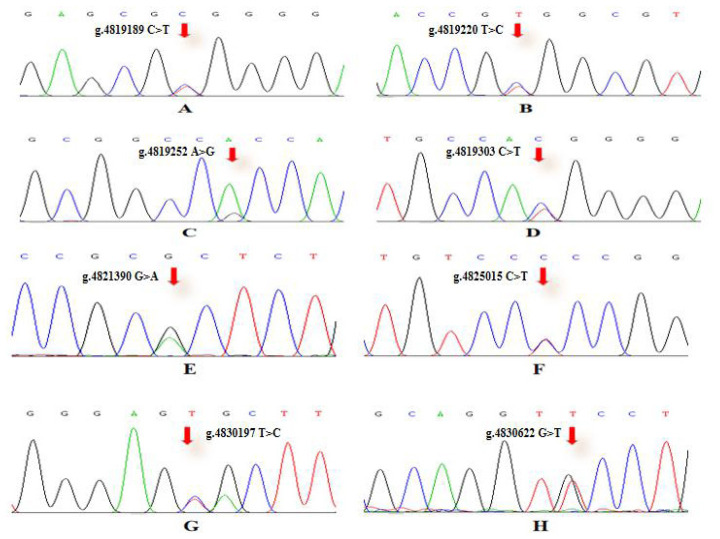
SNPs sequencing chromatogram of primer in *FKBP5* gene. (A) SNP locu of g.4819189 C>T. (B) SNP locu of g.4819220 T>C. (C) SNP locu of g.4819252 A>G. (D) SNP locu of g.4819303 C>T. (E) SNP locus of g.4821390 G>A. (F) SNP locu of g.4825015 C>T. (G) SNP locu of g.4830197 T>C. (H) SNP locu of g.4830622 G>T. The Chromas 1.62 software was used to detect *FKBP5* SNPs. SNPs, single nucleotide polymorphisms; *FKBP5*, FKBP prolyl isomerase 5.

**Figure 5:**
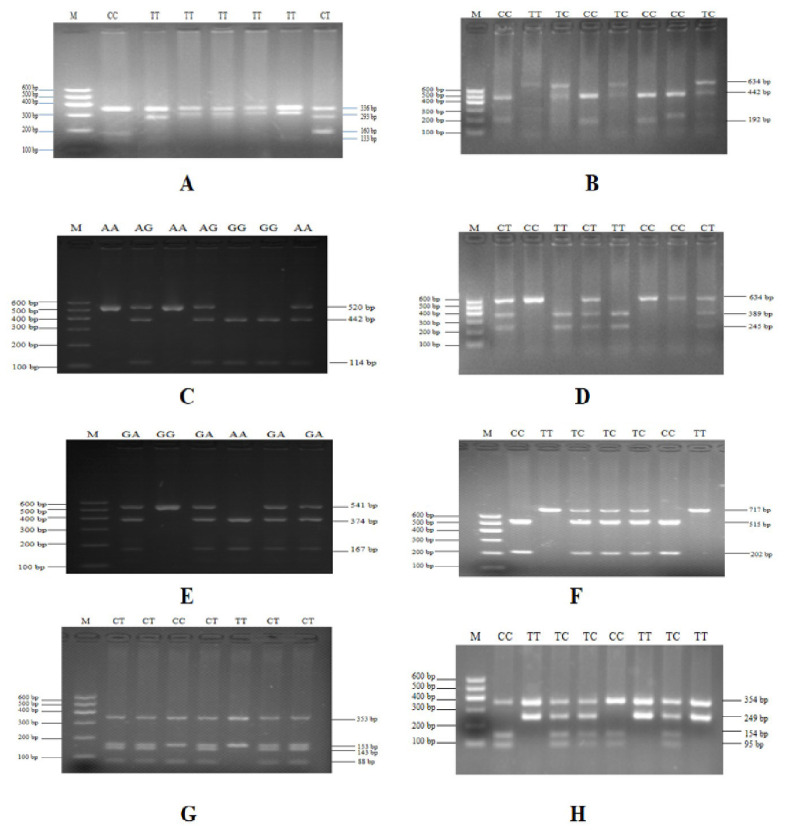
PCR-RFLP detection of the *FKBP5* gene PCR products. (A) Digestion of g. 4819189 C> T site by *Hha* I. (B) Digestion of g.4819220 T>C site by *Sac* II. (C) Digestion of g.4819252 A>G site by *Not* I. (D) Digestion of g.4819303 C>T site by *Nco* I. (E) Digestion of g.4821390 G>A site by *Hha* I. (F) Digestion of g.4825015 C>T site by *Sma* I. (G) Digestion of g.4830197 T>C site by *Hha* I. (H) Digestion of g.4830622 G>T site by *Ban* I. Lane M was DNA marker I. PCR-RFLP, polymerase chain reaction-restriction fragment length polymorphism; *FKBP5*, FKBP prolyl isomerase 5.

To verify the relationship between the SNP locus of *FKBP5* gene and duck body weight, blood samples were collected from 344 Muscovy ducks for DNA extraction. Then 9 pairs of primers were designed to amplify the duck *FKBP5* gene fragments (Figure 3). A total of 8 SNPs were identified by pool DNA or individual DNA sequencing, including g.4819189 C>T, g.4819220 T>C, g.4819252 A>G, g.4819303 C>T, g.4821390 G>A, g.4825015 C>T, g.4830197 T>C, and g.4830622 G>T (Figure 4), and the corresponding restriction enzymes are shown in Table 1. Eight SNP loci were detected by PCR-restriction fragment length polymorphism, as shown in Figure 5. In the sequence of S1, 4 mutation sites were found, and these SNPs which g. 4819189 C>T, g. 4819220 T>C, g. 4819252 A>G, g. 4819303 C>T were genotyped by the restriction enzyme *Hha* I (resulted in three banding patterns: digested genotypes CC, CT, and TT), *Sac* II (digested genotypes TT, TC, and CC), *Not* I (digested genotypes AA, AG, and GG) and *Nco* I (digested genotypes CC, CT, and TT), respectively. The PCR fragment of S4 in *FKBP5* had a restriction site, which g. 4821390 G>A was cut into GG, GA, and AA by *Hha* I. The sequence of S5 (g. 4825015 C>T) was cleaved into TT, TC, and CC by *Sma* I. Product of S8 (g. 4830197 T>C) was cut into CC, CT, and TT by *Hha* I. Besides, the mutation site of S9, which g. 4830622 C>T was digested into the genotype TT, CT, and CC by *Ban* I.

### Genetic diversity

The SNP allele and genotype frequency of *FKBP5* in Muscovy duck are shown in Table 2, the results showed a consensus that the C allele was dominant in 3 SNPs of g.4819189 C>T, g.4819303 C>T and g.4825015 C>T, which were 71.15%, 50.60%, and 56.85%, respectively. The A allele in g.4819252 A>G and g.4821390 G>A was dominant, accounting for 58.80% and 53.70%, respectively. Additionally, the frequencies of allele T were 81.80% (g.4819220 T > C), 78.20% (g.4830197 T>C) and 75.55% (g.4830622 G>T) in the other three SNP loci, respectively. The χ^2^ test results showed that all SNP sites were consistent with Hardy-Weinberg equilibrium. The diversity parameter He of Muscovy duck was between 0.2986 and 0.5891, and the Ne values were between 1.4256 and 1.9997. In addition, the values of PIC possessed intermediate polymorphism (0.25<PIC<0.5), which were between 0.2540 and 0.3750.

### Association analysis of single single nucleotide polymorphism loci

Relationships between the 8 SNPs of *FKBP5* and body weight trait were examined in 334 Muscovy ducks (Table 3). At the g.4819252 A>G (located in intron region), the genotype-AG individuals had greater body weight than the AA and the GG individuals (p<0.05), and the weight of GG genotype of Muscovy duck was significantly higher than that of the AA (p<0.05). At the g.4821390 G>A (located in intron region), genotype GA was extremely significantly related to body weight (p<0.01). Besides, at the g.4830622 T>G (located in 3′ UTR region), the TT type had a body weight that was significantly higher than the GG and the TG individuals (p<0.05), indicating that genotype TT was related to the body weight of Muscovy duck. In addition, other SNP loci were not associated with Muscovy duck weight (p>0.05). Haplotypes from the 344 individuals were analyzed using Haploview, and 8 haplotypes were found in g.4819252 A>G, g.4821390 G>A and g.4830622 T>G. AAG was the most common haplotype, with a frequency of 22.9%; next came AGG, GAG, and GGG, occurring with frequencies of 21.7%, 20.1%, and 10.9%, respectively (Table 4).

## DISCUSSION

### Tissue expression analysis of Muscovy duck *FKBP5*

Skeletal muscle plays a key role in the movement of the body to maintain vital activity. At the same time, skeletal muscle affects the energy metabolism of the whole body by regulating such as the fine energy production and consumption system. In animal husbandry, muscle production and muscle growth rate are factors that affect the important economic value of livestock and poultry. In addition to being closely related to breed and feeding condition [26], skeletal muscle development of animals is also associated with muscle-related regulatory genes. To date, there have been many studies on gene expression related to muscle development [27,28]. *FKBP5* gene is involved in cellular actions including regulation of cell proliferation, autophagy, osteoclast formation and insulin resistance in adipose tissue. In mice, *FKBP5* increased the mass of skeletal muscle, which may be through the enhancement of muscle protein synthesis and myotube differentiation, as well as inhibition of muscle protein degradation [29]. Therefore, we aimed to explore the expression of *FKBP5* in Muscovy duck tissues, as well as the relationship between *FKBP5* SNPs and body weight. In this study, there was a significant difference in *FKBP5* gene expression between the embryonic and adult Muscovy duck tissues. In addition, a significant relationship between SNPs and body weight characteristic in Muscovy duck population was found.

In the middle and late stages of embryo development, the tissues of Muscovy ducks developed rapidly. In our study, from the 17 d prior to hatching, the tissues were separated every 2 to 4 days, and the expression level of *FKBP5* was analyzed by qRT-PCR. Results of qRT-PCR indicated that *FKBP5* mRNA was widely expressed in different tissues of Muscovy duck. In E17d, E19d, E21d, E24d and E27d, the expression levels of *FKBP5* gene in Muscovy duck heart, liver, lung, breast muscle and leg muscle were significantly or extremely significantly higher than that of E31d (p<0.05 or p<0.01), while the expressions of *FKBP5* gene in kidney in E17d, E19d, E24d, and E27d were extremely significantly higher than those of the 31 d (p<0.01). In all tissues, the expressions of *FKBP5* gene in E34d were lower than that of E31d, but the differences were not statistically significant (p>0.05). It was indicated that the expression level of *FKBP5* had been reduced to a low level at the end of the embryonic period. In particular, in breast muscle of Muscovy duck, *FKBP5* was highly expressed in the middle stage of hatching, and it reached the highest point in E19d. It was speculated that the duck’s breast muscle was developing rapidly at this time. With the extension of incubation time, the expression of *FKBP5* gene gradually decreased to the lowest level after E31d. It was suspected that the duck’s breast muscle had basically developed. Similarly, the expression of *FKBP5* in leg muscle was highly expressed before E27d, and the expression was gradually reduced after E31d. The results showed that the Muscovy duck’s leg muscle developed rapidly before the 27 d of hatching, and the development of leg muscle was nearing completion after 31 d of the incubation. *FKBP5* plays an important role in regulating the individual metabolism [15,30]. Compared with other tissues, the high expression level of *FKBP5* in metabolically active tissues (such as skeletal muscle and adipose tissue) further supports the role of *FKBP5* in systemic metabolism [17]. In the embryonic stage of Muscovy duck, the tissues such as heart, liver, lung, kidney, and skeletal muscle developed rapidly and metabolized vigorously, which may be one of the reasons for the high expression of *FKBP5*. Therefore, *FKBP5* may promote the growth and development of Muscovy duck, especially skeletal muscle, at the middle and late stages of embryo development. Interestingly, in the tissues of adult female Muscovy ducks, the expressions of *FKBP5* were always higher than that of male Muscovy ducks (except liver). More importantly, the expression of *FKBP5* in leg muscle of female Muscovy ducks was significantly higher than male ducks. In addition, the gene expression in ovary was significantly higher than that of testis. It could be explained by the fact that FKBP51, as part of the heat shock protein 90 steroid receptor complex, plays an important role in regulating the steroid hormone receptor (GR), which involves a wide range of physiological metabolic processes including immune, cardiovascular, reproductive, neurological, and metabolic systems [31]. Due to egg production and other factors (such as follicle development), the physiology and metabolism of female ducks are higher than that of males [32]. Therefore, the expressions of *FKBP5* in female Muscovy duck tissues were higher than that in male Muscovy duck.

### Association between *FKBP5* gene and Muscovy duck weight

So far, there were few studies about the association analysis between SNPs of *FKBP5* and body weight. Therefore, we mainly focused on mutations of *FKBP5* in Muscovy duck and studied whether mutations of *FKBP5* affected the body weight. By sequencing the PCR products, 8 SNP sites were found. Then enzyme digestion on these SNP sites was performed, and statistical analysis was conducted. The χ^2^ test results showed that all SNP loci conformed to the Hardy-Weinberg equilibrium law. It meant that the frequency of each allele was stable. In other words, the genetic balance was maintained. The genetic parameters He and Ne of the SNP loci in Muscovy duck population ranged from 0.2986 to 0.5891 and 1.4256 to 1.9997, respectively. PIC value represented the level of genetic diversity (PIC value <0.25, low genetic diversity; 0.25<PIC value<0.50, intermediate genetic diversity; and PIC value >0.50, high genetic diversity), the values of PIC were in the range of 0.2540 to 0.3750, which were a moderate polymorphic range, indicating that each SNP locus was more polymorphic in Muscovy duck population.

Finally, 3 SNP loci (g.4819252 A>G, g.4821390 G>A, and g.4830622 T>G) were found in Muscovy duck *FKBP5* gene related to body weight, and haplotype results showed that the frequencies of AAG, AGG, GAG, and GGG were higher. Because muscle accounts for 50% to 60% of body weight, we believe that these SNP sites may regulate muscle development and increase muscle mass. Specifically, genotype AG of g.4819252 A>G, genotype GA of g.4821390 G>A and genotype TT of g.4830622 T>G were highly correlated with Muscovy duck weight. Notably, g.4819252 A>G and g.4821390 G>A are in the intron of *FKBP5* gene. The transcription efficiency of genome may be directly affected by the base mutation in intron region of gene. Six intron SNPs of *ECHS1* were significantly associated with milk fatty acids in dairy cows [33]. Cao et al [34] found that SNP sites in introns may change the formation and occurrence of the spliceosome, and ultimately affect protein biosynthesis. In addition, g.4830622 T>G is in the 3′ UTR region of *FKBP5*, and the 3′UTR SNP can regulate the post-transcriptional gene expression by affecting gene transcription activity, mRNA splicing, stability of mRNA and the binding of miRNA to target mRNA [35]. Ju et al [36] suggested that the SNP g.18475 A>G in 3′UTR of neutrophil cytosolic factor 4 (*NCF4*) was associated with mastitis susceptibility in dairy cows. Therefore, our results suggested that *FKBP5* could see potential use as a molecular marker in future breeding programs to increase growth rates of Muscovy ducks.

## CONCLUSION

In this study, the expressions of *FKBP5* gene were detected in embryonic and adult Muscovy duck tissues. The results showed that *FKBP5* was significantly expressed in different tissues at the stages of embryonic development. In addition, the expressions of *FKBP5* in adult female duck tissues were higher than that of male ducks. Subsequently, the correlation between Muscovy duck *FKBP5* SNPs and body weight was studied. It was found that 3 SNPs (g.4819252 A>G, g.4821390 G>A, and g.4830622 T>G) were significantly associated with Muscovy duck weight. These findings indicated that *FKBP5* could be used as a molecular marker for muscle development traits by early marker-assisted selection of Muscovy duck.

## Figures and Tables

**Table 1:** qRT-PCR and PCR Primers for duck *FKBP5* gene

Name	Primers (5′→3′)	Fragment size (bp)	Restriction enzyme^1),2)^
RT-*FKBP5*	Forward primer TCCAACGCCACCCTCTTC	193	
	Reverse primer ACTTCACGTCCTTGCAGTCG		
RT-*β-actin*	Forward primer CCCTGTATGCCTCTGGTCG	194	
	Reverse primer CTCGGCTGTGGTGGTGAAG		
S1	Forward primer TCCAGCTTTTAACCCTTTCC	634	*Hha* I (336, 293, 160, 133, 5), Sac II (634, 442, 192)
	Reverse primer CGTTTCACTACTCGCTTCTTGT		*Not* I (520, 442, 114), Nco I (634, 389, 245)
S2	Forward primer CCTGCGAGTGAAATCTGC	609	None
	Reverse primer GATGTCCCACGCCTTGAT		
S3	Forward primer GCACTACAAAGGCAAACTG	777	None
	Reverse primer AGCACCTGAAGCCCAACA		
S4	Forward primer GTGGCTTCCCCGTTGTTT	541	*Hha* I (541, 374, 167)
	Reverse primer GACGCAGGAGGGGTAAAT		
S5	Forward primer TAATCGCCGTGATAAACTCC	717	*Sma* I (717, 515, 202)
	Reverse primer CCTTCCACCGAGGCTAAA		
S6	Forward primer AGCCCCTCAAACTCAACG	655	None
	Reverse primer TTTCCAGCCAGGACACGA		
S7	Forward primer CTGGCGAACTCTGTCCTT	387	None
	Reverse primer ACCTCAGCATCCTCAACCC		
S8	Forward primer TTGCCAGGACTCAAGGAC	803	*Hha* I (353, 153, 143, 88, 66, 55)
	Reverse primer CCCAGCTAAGCGATAAAAC		
S9	Forward primer AGGCGTTTCTCATCCGTG	648	*Ban* I (354, 249, 154, 95, 45)
	Reverse primer GCTGAGATCCCAAACAAC		

qRT-PCR, real-time quantitative PCR; *FKBP5*, FKBP prolyl isomerase 5.

1) The “None” means that there is no SNP site on the amplified fragment or there is no suitable restriction enzyme in the SNP site.

2) The numbers in brackets are the length of fragment after endonuclease digestion (bp).

**Table 2:** Allele and genotype frequencies of *FKBP5* SNP locus of Muscovy duck

SNP locus	Genotype frequency/genotype			Allele frequency		Diversity parameter			

						χ^2^	He	Ne	PIC
	
g.4819189 C>T	P_CC_	P_CT_	P_TT_	P_C_	P_T_				
	0.593/198	0.237/79	0.171/57	0.7115	0.2885	p>0.05	0.5891	1.6975	0.3265
g.4819220 T>C	P_TT_	P_TC_	P_CC_	P_T_	P_C_				
	0.716/239	0.204/68	0.081/27	0.8180	0.1820	p>0.05	0.2986	1.4256	0.2540
g.4819252 A>G	P_AA_	P_AG_	P_GG_	P_A_	P_G_				
	0.275/92	0.626/209	0.099/33	0.5880	0.4120	p>0.05	0.4844	1.9395	0.3671
g.4819303 C>T	P_CC_	P_CT_	P_TT_	P_C_	P_T_				
	0.449/150	0.114/38	0.437/146	0.5060	0.4940	p>0.05	0.4999	1.9997	0.3750
g.4821390 G>A	P_GG_	P_GA_	P_AA_	P_G_	P_A_				
	0.111/37	0.704/235	0.186/62	0.4630	0.5370	p>0.05	0.4972	1.9889	0.3736
g.4825015 C>T	P_CC_	P_CT_	P_TT_	P_C_	P_T_				
	0.383/128	0.371/124	0.254/85	0.5685	0.4315	p>0.05	0.4905	1.9628	0.3702
g.4830197 T>C	P_TT_	P_TC_	P_CC_	P_T_	P_C_				
	0.701/234	0.162/54	0.138/46	0.7820	0.2180	p>0.05	0.3416	1.5188	0.2832
g.4830622 G>T	P_GG_	P_GT_	P_T T_	P_G_	P_T_				
	0.150/50	0.189/63	0.661/221	0.2445	0.7555	p>0.05	0.3689	1.5846	0.3009

*FKBP5*, FKBP prolyl isomerase 5; SNP, single nucleotide polymorphism; He, the gene heterozygosity; Ne, the effective allele numbers; PIC, polymorphism information content.

**Table 3:** Association of different SNP genotypes with body weight in Muscovy duck

SNPs	Genotype	Body weight (kg)	Restriction enzyme	Location
g.4819189 C>T	CC (n = 198)	3.15±0.65	*Hha* I	Exon
	CT (n = 79)	3.20±0.50		
	TT (n = 57)	3.05±0.55		
	p-value	p>0.05		
g.4819220 T>C	TT (n = 239)	3.05±0.80	*Sac* II	Exon
	TC (n = 68)	3.10±0.65		
	CC (n = 27)	2.95±0.50		
	p-value	p>0.05		
g.4819252 A>G	AA (n = 92)	2.75^c^±0.55	*Not* I	Intron
	AG (n = 209)	3.25^a^±0.65		
	GG (n = 33)	3.00^b^±0.60		
	p-value	p<0.05		
g.4819303 C>T	CC (n = 150)	3.05±0.75	*Nco* I	Intron
	CT (n = 38)	3.10±0.60		
	TT (n = 146)	3.15±0.65		
	p-value	p>0.05		
g.4821390 G>A	GG (n = 37)	2.75^B^±0.65	*Hha* I	Intron
	GA (n = 235)	3.15^A^±0.50		
	AA (n = 62)	2.70^B^±0.65		
	p-value	p<0.01		
g.4825015 C>T	CC (n = 128)	2.95±0.55	*Sma* I	Intron
	CT (n = 124)	3.10±0.65		
	TT (n = 85)	3.05±0.55		
	P-value	p>0.05		
g.4830197 T>C	TT (n = 234)	3.05±0.55	*Hha* I	3′UTR
	TC (n = 54)	2.90±0.60		
	CC (n = 46)	2.85±0.55		
	p-value	p>0.05		
g.4830622 T>G	TT (n = 50)	3.25^a^±0.75	*Ban* I	3′UTR
	TG (n = 63)	3.00^b^±0.60		
	GG (n = 221)	2.85^b^±0.65		
	p-value	p<0.05		

The values in the table are mean±standard deviation.

SNP, single nucleotide polymorphism.

A,B, a–c In the same column, lowercase letter indicates significant differences (p<0.05), uppercase letter indicates significant differences (p<0.01), and the same letter indicates the difference was not significant (p>0.05).

**Table 4:** Haplotypes of *FKBP5* gene and their frequencies in Muscovy duck

Haplotype	g.4819252 A>G	g.4821390 G>A	g.4830622 T>G	Frequency
Hap1	A	A	G	0.229
Hap2	A	A	T	0.056
Hap3	A	G	G	0.217
Hap4	A	G	T	0.086
Hap5	G	A	G	0.201
Hap6	G	A	T	0.051
Hap7	G	G	G	0.109
Hap8	G	G	T	0.050

*FKBP5*, FKBP prolyl isomerase 5.

## SUPPLEMENTARY MATERIAL

The feed composition of adult Muscovy duck
