# Supplementary material for: Expression of FKBP prolyl isomerase 5 gene in tissues of muscovy duck at different growth stages and its association with muscovy duck weight
Source: Anim Biosci. 2021 Jun 23;35(1):1–12. doi: 10.5713/ab.20.0649 (PMC8738923; doi:10.5713/ab.20.0649)
Supplement: Supplementary file 1 [file ab-20-0649-suppl.pdf]

Table S1. The feed composition of adult Muscovy duck

Ingredient	Content (%)	Nutrient	Content (%)
Corn	56.00	Crude protein	15.700
Soybean meal	23.80	Calcium	0.900
Corn gluten meal	10.00	Total phosphorus	0.680
Limestone	7.00	Available phosphorus	0.450
CaHPO4	1.50	Salt	0.370
Premix	1.00	Lysine	0.760
NaCl	0.30	Methionine	0.387
Lys·HCl	0.30	Methionine + Cystine	0.654
DL-Met	0.10	Isoleucine	0.534
Total	100.00	Threonine	0.579
		Tryptophan	0.194
		Crude fiber	4.100
		Crude fat	3.400
		Crude ash	5.200
		Avian metabolizable energy	2875 Mcal·kg-1
